# Preventing sight loss from maculopathy

**Published:** 2015

Laser treatment is less successful at reducing the risk of diabetic macular oedema (DMO) causing visual loss compared to peripheral retinal photocoagulation for proliferative diabetic retinopathy. Intravitreal treatments are effective but often unavailable due to cost and access to treatment.

## When to do macular laser

The threshold for macular laser is usually clinically significant macular oedema (CSMO) as defined in the Early Treatment of Diabetic Retinopathy Study (ETDRS). CSMO is any retinal thickening (oedema) and/or exudates within 500 microns (⅓ disc diameter) of the centre of the fovea; or oedema greater than 1 disc area within 1 disc diameter of the foveal centre – including oedema which involves the fovea already. Macular laser is more effective if the DMO is localised (focal maculopathy), than if it is generalised across the central macula (diffuse maculopathy). Optical Coherence Tomography (OCT) scans enable visualisation of macular oedema in great detail, but are not required to determine whether a patient meets the thresholds for macular laser, because these were determined prior to the advent of OCT.

Exudates, if they involve the fovea, can sometimes threaten or affect vision without macular oedema. Exudates without oedema within 500 microns of the foveal centre, particularly long or streak exudates pointing towards the centre, are an indication for laser.

## Tips for successful macular laser

**Figure 3.**
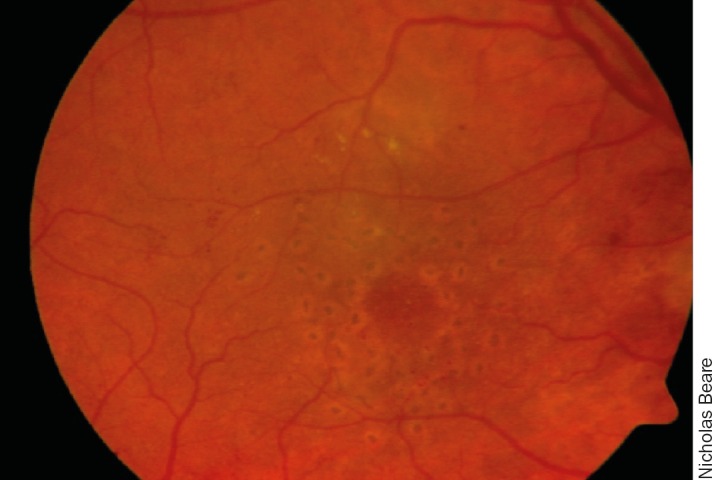
An example of macular grid laser around the fovea. There is potential to extend this outwards if necessary, but not any closer to the centre of the fovea
- Most operators like to start with the inner ring after titrating the laser power, and then move outwards to complete 4–6 rings. This should create an adequate grid to treat a diffuse maculopathy.
- If further treatment is required, and the patient can keep still, a further ring, 500 microns (⅓ disk diameter) from the centre, can be added. However, once the grid is complete there is nothing to be gained by further grid laser. This is unlike PRR, where fill-in laser can be repeated many times if necessary.
- When targeting microaneurysms, the ideal result from laser is a colour change. However, it is difficult to land a direct hit with one shot, and not more than 5 attempts should be made on an individual microaneurysm. As microaneurysms occur in the thickened retina, it may be necessary to focus slightly anteriorly to treat them accurately.
- The spaces between burns for the grid laser should be approximately the width of 1 spot.

Macular laser is much more gentle and measured than peripheral retinal photocoagulation (PRP). Macular laser can be directed at microaneurysms, or applied in a grid pattern over the oedematous zone. Macular laser is usually given as a combination of both, which is known as a Modified Macular Grid: the microaneurysms are targeted first, and untreated areas of oedema are then treated in a grid pattern (see Figure 3).

Although it is important to make sure that the laser beam is focused in PRP, this is **critical** in macular laser. Retinal lasers are not in a parallel beam but converge to a focus. Whilst setting up the laser, make sure that the laser focus and the optical focus of the slit lampare in the same plane. Lasers are usually supplied with a test rod, but the focus adjustment can be done on the fundus with the laser in standby. Focus the aiming beam, and then adjust each eyepiece until the view is also in focus.

For macular laser:

- The default laser spot size is 100 microns; however, smaller sizes can be used if small microaneurysms are being targeted and the patient is very still.
- Short durations, such as 0.02 s, result in less retinal damage but may need to be increased for microaneurysm treatment.
- The power should be low to start with (100 mW) and increased in steps of 50 mW as necessary (the same as for PRP). However, the desired burn should be just visible (less visible than for PRP).
- Titrate the laser power away from the fovea, on the edge of the macular oedema. This is because uptake is less good within zones of oedema. It may be necessary to increase the power in zones of oedema, but limit this to an increase of 100mW.
- For a first macular laser treatment, the laser burns should be 750 microns (half a disc diameter) from the centre of the fovea. If the fovea is hard to discern then the inner ring should be wider, outside the zone including the fovea.

Macular laser takes 8 weeks to a year to have its full effect so do not rush to judge it. The main aim is to prevent deterioration of vision, and the recent trials with a laser cohort show that stable vision is about what is achieved on average. However, for focal maculopathy with good vision, maintenance of that good vision with laser is well worthwhile.

## Complications of macular laser

The complication to be avoided in macular laser is foveal burn. This can occur if:

1. Microaneurysms closer than 500 microns (⅓ disc diameter) from the foveal centre are targeted.
2. If care is not taken to avoid the whole central macula when the position of the fovea is unclear.
3. If the patient moves suddenly.

The worst scenario is immediate loss of central vision, but with short laser durations this can be mitigated. Sometimes patients are aware of paracentral scotomas from the laser burns if they are close to the foveal centre – it is therefore important to listen to what they have to say.

## Role of intravitreal treatment in maculopathy

Recent studies have shown that anti-VEGF treatment produces greater visual improvements than laser in patients with central diabetic macular oedema (DMO) whose vision is reduced to 6/12 or worse. These intravitreal injections reduce DMO rapidly and effectively. Laser treatment usually prevents loss of vision but does not often lead to visual improvement. Repeated injections of bevacizumab in eyes with visual acuity of less than 6/12 give an average improvement of two lines on the Snellen chart, and about a quarter of patients will improve by three lines. However, they have a number of problems – notably cost, the treatment burden of monthly injections, and the risk of infection/endophthalmitis. Even the relatively low cost of bevacizumab is prohibitive to many patients in low- and middle-income countries. Furthermore, a reliable pharmacy is required, one which can divide the intravenous dose into intravitreal doses in sterile conditions. Clusters of endophthalmitis cases in the US and UK have led to a suspicion of contaminated batches of anti-VEGF preparations. This is a definite concern in less well-regulated areas.

Evidence from clinical trials suggests that patients require 9–12 injections in the first year, so the treatment regime is intense – requiring frequent revisits in order to maximise the benefits. After the first year, the overall treatment burden is less, but some patients have recurring DMO requiring ongoing retreatments.

Despite these problems, the greater effectiveness of intravitreal injections means that they may be valuable for some patients, particularly those who can afford the drug costs and live sufficiently near to the clinic to attend for repeated treatment.

An alternative intravitreal treatment is steroid, the least costly being triamcinolone. This is effective, but visual gains are reduced by induced cataract, and even after cataract surgery on average the vision does not catch up with that from anti-VEGF therapy. This may be due to exacerbated DMO or postoperative cystoid macular oedema. Intravitreal steroid is an option, especially in patients who are already pseudophakic. Post-injection intraocular pressure (IOP) rise can be a problem, so this needs to be monitored and treated accordingly.

